# First instalment in resolution of the *Banksia spinulosa* complex (Proteaceae): *B. neoanglica*, a new species supported by phenetic analysis, ecology and geography

**DOI:** 10.3897/phytokeys.14.3415

**Published:** 2012-08-03

**Authors:** Margaret L. Stimpson, Peter H. Weston, Ian R.H. Telford, Jeremy J. Bruhl

**Affiliations:** 1 Botany, School of Environmental and Rural Science and N.C.W. Beadle Herbarium, University of New England, Armidale NSW 2351 Australia; 2 National Herbarium of New South Wales, The Royal Botanic Gardens and Domain Trust, Mrs Macquaries Road, Sydney, NSW 2000, Australia

**Keywords:** *Banksia spinulosa*, *Banksia cunninghamii*, *Banksia neoanglica*, species limits, phenetics, new species, floral and inflorescence morphology

## Abstract

Taxa in the *Banksia spinulosa* Sm. complex (Proteaceae) have populations with sympatric, parapatric and allopatric distributions and unclear or disputed boundaries. Our hypothesis is that under biological, phenetic and diagnosable species concepts that each of the currently named taxa within the *Banksia spinulosa* complex is a separate species. Based on specimens collected as part of this study, and data recorded from specimens in six Australian herbaria, complemented by phenetic analysis (semi–strong multidimensional scaling and UPGMA clustering) and a detailed morphological study, we investigated both morphological variation and geographic distribution in the *Banksia spinulosa* complex. All specimens used for this study are held at the N.C.W. Beadle Herbarium or the National Herbarium of New South Wales. In total 23 morphological characters (11 quantitative, five binary, and seven multistate characters) were analysed phenetically for 89 specimens. Ordination and cluster analysis resulted in individuals grouping strongly allowing recognition of distinct groups consistent with their recognition as separate species. Additional morphological analysis was completed on all specimens using leaf, floral, fruit and stem morphology, providing clear cut diagnosable groups and strong support for the recognition of *Banksia spinulosa* var. *cunninghamii* and *Banksia spinulosa* var. *neoanglica* as species.

## Introduction

*Banksia* is a moderately sized genus currently of 212 taxa; viz. 78 species, 9 subspecies and 11 varieties (see Collins et al. 2009), plus 114 species previously included under *Dryandra* (Mast and Thiele 2007). There are16 named species of *Banksia* in the eastern states of Australia (Collins et al. 2009). Species of *Banksia* are often found in sclerophyllous, heathy shrublands on nutrient poor soils and have spectacular spike-like cylindrical or flattened, head-like conflorescences that are easily recognised (Mast et al. 2005). The growth habit in *Banksia* ranges from small prostrate woody mats to 25 m tall trees. Only one species occurs naturally outside Australia, *Banksia dentata*, which extends to Papua New Guinea, Irian Jaya and the Aru Islands (George 1981; Mast et al. 2002).

According to George’s (1999) classification, the *Banksia spinulosa* complex has the broadest latitudinal, altitudinal and ecological amplitude of any species in the genus (Thiele and Ladiges 1996). The *Banksia spinulosa* complex consists of four taxa distributed from north-eastern Queensland to eastern Victoria along the coast and highlands. *Banksia spinulosa* var. *spinulosa* occupies both latitudinal extremes but is replaced along the coast between the Sunshine Coast area in south-eastern Queensland and the Hawkesbury River in central eastern New South Wales by *Banksia spinulosa* var. *collina*, which also has inland outliers west to the Carnarvon National Park area.* Banksia spinulosa* var. *cunninghamii* is mostly a montane taxon distributed mostly between the Hunter River in central eastern New South Wales and eastern Victoria, with a northern disjunction in the McPherson Range along the Queensland–New South Wales border. It is broadly sympatric with, and frequently co-occurs in mixed populations alongside *Banksia spinulosa* var. *spinulosa* between the northern Blue Mountains and the Moss Vale district. *Banksia spinulosa* var. *neoanglica* is also a montane taxon, distributed from the McPherson Range and along the eastern edge of the New England Tableland, New South Wales south to the Hanging Rock area. *Banksia spinulosa* var. *neoanglica* is parapatric with a montane variant currently attributed to *Banksia spinulosa* var. *collina* in the Daves Creek area, Lamington National Park; it is allopatric with other taxa in the complex.

Most herbaria follow George (1981, 1988, 1999) in treating this complex as one species with four varieties, viz. *Banksia spinulosa* var. *spinulosa*, *Banksia spinulosa* var. *collina*, *Banksia spinulosa* var. *cunninghamii*, and *Banksia spinulosa* var. *neoanglica*. Flora of New South Wales (NSW) Online (1999 onwards) treats the *Banksia spinulosa* complex as comprising two species, each with two infraspecific taxa: *Banksia spinulosa* var. *collina*, *Banksia spinulosa* var. *spinulosa*, *Banksia cunninghamii* subsp. *cunninghamii*, and *Banksia cunninghamii* subsp. A (= *Banksia spinulosa* var. *neoanglica*), and this paper will use this treatment as a reference point. The primary reason for recognising two species was the broad sympatry of *Banksia spinulosa* var. *spinulosa* and *Banksia cunninghamii* subsp. *cunninghamii*. There appearsto be no hybridisation between these two taxa, indicating that these two taxa are reproductively isolated from one another and are therefore different biological species (Harden 2002).

These competing taxonomic treatments have created confusion, examples of which can be found in species lists for some National Parks in New South Wales (unpublished visitor brochures), which include *Banksia spinulosa* var. *neoanglica* and *Banksia cunninghamii* subsp. A as separate entities. Some herbaria also concurrently use two names for the same entity (see the Atlas of Living Australia). Current circumscriptions of the taxa within the *Banksia spinulosa* complex are based on intuitive assessment of observed morphological variation, rather than an explicit analysis of the morphological variation. Thiele and Ladiges (1996) conducted a cladistic analysis of the whole of *Banksia* using 92 qualitative characters and 14 morphometric characters in an attempt to clarify interspecific relationships and to provide a phylogenetic classification. As that was a genus-wide study, limited work was conducted on or within individual species.

The aims of this study were (1) to test and set the taxonomic status and circumscription of *Banksia cunninghamii* subsp. A; and (2) to search for novel diagnostic characters that could be used to distinguish individual taxa within the *Banksia spinulosa* complex (*sensu* George 1988).

## Materials and methods

### Study material

Although dried herbarium specimens were available for this study, it was considered necessary to collect fresh material to adequately investigate character homology though a detailed study of different developmental stages. Existing herbarium specimens were deficient in some developmental stages and often were not suitable for destructive sampling. We made collections from locations in New South Wales and Queensland encompassing the full geographic range of *Banksia cunninghamii* subsp. A. Vouchers have been lodged at NE and/or NSW (Table 1). Each site was visited twice; the first time in February to observe the development of the rachis, the second time in May to observe the flowering process. During both visits observations were made and vouchers prepared.

**Table 1.** Vouchers used in phenetic and morphological analysis of the *Banksia spinulosa* complex.Numbers in the OTU code are M. L. Stimpson collection numbers. Bcu = *Banksia cunninghamii* subsp. *cunninghamii*; Bco = *Banksia spinulosa* var.* collina*; Bn = *Banksia cunninghamii* subsp. A; Bsp = *Banksia spinulosa* var. *spinulosa*; Bsp? = putative hybrid of *Banksia spinulosa* var. *collina* **×** *Banksia spinulosa* var. *spinulosa*. Abbreviations: NP = National Park; NSW = New South Wales; Qld = Queensland. Voucher codes are herbarium abbreviations following Thiers (continuously updated). All elements of the collections were available at NE and/or NSW during the study, replicates will be distributed.

**OTU Code**	**Location**	**Voucher**
BcuHW30	Hassans Walls, Hartley Vale, NSW	NE, NSW
BcuHW32	Hassans Walls, Hartley Vale, NSW	NE, NSW
BcuHW41	Hassans Walls, Hartley Vale, NSW	NE, NSW
BcuSR43a	Scenic Railway, Katoomba, NSW	NE, NSW
BcuSR43b	Scenic Railway, Katoomba, NSW	NE, NSW
BcuSR43c	Scenic Railway, Katoomba, NSW	NE, NSW
BcuHW114	Hassans Walls, Hartley Vale, NSW	NE, NSW
BcuHW115	Hassans Walls, Hartley Vale, NSW	BRI, NE, NSW
BcuEL117	Evans Lookout, Blue Mountains, NSW	BRI, NE, NSW
BcuCF118	Cataract Falls, Blue Mountains, NSW	NE, NSW
BcuML119	McMahons Lookout, Blue Mountains, NSW	NE, NSW
BcuFF121	Fitzroy Falls, E of Moss Vale, NSW	NE, NSW
BcuFF128	Fitzroy Falls, E of Moss Vale, NSW	NE, NSW
BcuMW122	Medway, W of Moss Vale, NSW	NE, NSW
BcuMW123	Medway, W of Moss Vale, NSW	BRI, NE, NSW
BcuMW126	Medway, W of Moss Vale, NSW	NE, NSW
BcoK25a	Kungala, NSW	NE
BcoK25c	Kungala, NSW	NE
BcoK60a	Kungala, NSW	NE, NSW
BcoK60b	Kungala, NSW	NE
BcoK60c	Kungala, NSW	NE, NSW
BcoKR61	Kremnos, NSW	NE
BcoKR62	Kremnos, NSW	NE, NSW
BcoTG88	Tarragindi, Brisbane, Qld	NE, NSW
BcoDCK102	Daves Creek track, Lamington NP, Qld	NE
BcoDCK85	Daves Creek track, Lamington NP, Qld	NE, NSW
BcoDCK103	Daves Creek track, Lamington NP, Qld	NE
BcoMY93	Mullaway, NSW	NE
BnRN27a	Robinsons Knob Trail, New England NP, NSW	NE, NSW
BnRN27b	Robinsons Knob Trail, New England NP, NSW	NE, NSW
BnRN27c	Robinsons Knob Trail, New England NP, NSW	NE, NSW
BnBP28a	Banksia Point, New England NP, NSW	NE
BnBP28b	Banksia Point, New England NP, NSW	NE
BnBP28c	Banksia Point, New England NP, NSW	NE, NSW
BnNE39a	Point Lookout road, New England NP, NSW	NE
BnNE39b	Point Lookout road, New England NP, NSW	NE, NSW
BnNE39c	Point Lookout road, New England NP, NSW	NE, NSW
BnDCK79	Daves Creek track, Lamington NP, Qld	NE, NSW
BnDCK80	Daves Creek track, Lamington NP, Qld	NE, NSW
BnDCK81	Daves Creek track, Lamington NP, Qld	NE, NSW
BnDCK82	Daves Creek track, Lamington NP, Qld	NE
BnBP96	Banksia Point, New England NP, NSW	BRI, NE, NSW
BnTC97	Tom’s Cabin, New England NP, NSW	BRI, NE, NSW
BnMM98	Mount Mitchell, NSW	BRI, NE, NSW
BnMM99	Mount Mitchell, NSW	BRI, NE, NSW
BnG100	Girraween NP, Qld	BRI, NE, NSW
BnG101	Girraween NP, Qld	BRI, NE, NSW
BnDCK104	Daves Creek track, Lamington NP, Qld	NE
BnDCK105	Daves Creek track, Lamington, NP, Qld	NE, NSW
BnBB106	Boonoo Boonoo NP, Morgan’s Gully, NSW	BRI, NE, NSW
BnBB107	Boonoo Boonoo NP, Morgan’s Gully, NSW	NE, NSW
BnBB108	Boonoo Boonoo NP, Morgan’s Gully, NSW	NE, NSW
BnBB109	Boonoo Boonoo NP, Cyprus Pine Camp, NSW	NE, NSW
BnGR110	Gibraltar Range NP, Mulligans Hut, NSW	BRI, NE, NSW
BnGR111	Gibraltar Range NP, Mulligans Hut, NSW	NE
BnGR112	Gibraltar Range NP, Mulligans Hut, NSW	BRI, NE, NSW
BnGR113	Gibraltar Range NP, Mulligans Hut, NSW	NE
BspDC42	Darling Causeway, Blue Mountains, NSW	NE, NSW
BspHB44	Hazelbrook, Blue Mountains, NSW	NE, NSW
BspHB45	Hazelbrook, Blue Mountains, NSW	NE, NSW
BspJB46	Jervis Bay, NSW	NE, NSW
BspJB59	Jervis Bay, NSW	NE, NSW
BspML120	McMahons Lookout, Blue Mountains, NSW	NE, NSW
BspFF127	Fitzroy Falls, E of Moss Vale, NSW	NE, NSW
BspMW129	Medway, W of Moss vale, NSW	NE, NSW
BspML130	McMahons Lookout, Blue Mountains, NSW	NE, NSW
BspEL131	Evans Lookout, Blue Mountains, NSW	NE
BspCF132	Cataract Falls, Blue Mountains, NSW	NE, NSW
Bsp?BU36	Bouddi NP, NSW	NE, NSW
Bsp?BU37	Bouddi NP, NSW	NE
Bsp?BU38	Bouddi NP, NSW	NSW
Bsp?CA52a	Calga, NSW	NSW
Bsp?CA52b	Calga, NSW	NE, NSW
Bsp?CA52c	Calga, NSW	NE, NSW
Bsp?CA53a	Calga, NSW	NE, NSW
Bsp?CA53b	Calga, NSW	NE, NSW
Bsp?CA53c	Calga, NSW	NE, NSW
Bsp?M54a	Morisset, NSW	NE, NSW
Bsp?M54b	Morisset, NSW	NE, NSW
Bsp?M54c	Morisset, NSW	BRI, NE, NSW
Bsp?RM66	Morisset, NSW	NE, NSW
Bsp?YM67	Morisset, NSW	NE, NSW
Bsp?JB124	Jervis Bay, NSW	NE
Bsp?MM86	Mount Mee, Qld	NE
Bsp?MM87	Mount Mee, Qld	NE
Bsp?GM89	Glasshouse Mountains, Qld	NE
Bsp?GM90	Glasshouse Mountains, Qld	NE
Bsp?GM91	Glasshouse Mountains, Qld	NE
Bsp?GM92	Glasshouse Mountains, Qld	NE

### Observations and microscopy

Micromorphology was examined using Leica MZ8 and MZ9 stereomicroscopes fitted with eyepiece graticules. Images were taken using a Wild M400 photomacroscope fitted with a Nikon DS-5M-L1 Digital Sight Camera System. Exploratory scanning electron microscopy of styles was undertaken using air and silca gel-dried samples mounted on double-sided carbon tabs on aluminium stubs. Specimens were coated with gold in a Neocoater sputter coater and examined at 15 kV using a Neoscope JCM-5000 bench-top SEM.

## Phenetic analysis

### Selection of characters

The character list was primarily constructed to include leaf, floral, stem and fruit morphology. Assessment of descriptions of the taxa in the *Banksia spinulosa* complex (George 1981, 1988; Thiele and Ladiges 1996; Harden 2002) led to the selection of characters for the inclusion in the phenetic analysis. Additional characters were considered based on observed differences in the field (Table 2). Wherever possible, quantitative characters were used to reduce subjectivity and to avoid artefacts resulting from the conversion of continuous variables into categorical ones. Qualitative character states were scored as either 1 or 2. Quantitative characters for each OTU were the mean of up to 10 measurements where possible.

**Table 2.** Characters used for phenetic analysis for the *Banksia spinulosa* species complex.

**No.**	**Character and states**
	**Quantitative characters**
1	Length of complete conflorescence including peduncle ± 1 mm
2	Width of lamina at widest point excluding teeth ± 1 mm
3	Length of lamina including mucro ±1 mm
4	Length from base of lamina to first tooth excluding mucro ± 1 mm
5	Length of seed including wing ± 1 mm
6	Width of wing at widest point ± 1 mm
**7**	Length of seed excluding wing ± 1 mm
17*	Number of floral pairs around circumference of conflorescence
9	Length of complete infructescence ± 1 mm
10	Circumference of complete infructescence ± 1 mm
12	Lamina interveinal thickness when dry ± 0.05 mm
	**Binary characters**
11	Lignotuber: 1 = absent 2 = present
20*	Floral bract keel number: 1 = 1 2 = 2
21*	Distal bract margins: 1= plain 2 = recurved
22*	Bract apiculum: 1= absent 2 = present
23*	Bract apiculum: 1 = not incurved 2= incurved
	**Multistate characters**
8	Lamina apex: 1 = tridentate, 2 = bidentate, 3 = unidentate
13	Colour of lamina adaxial surface when dry^~^
14	Colour of lamina abaxial surface when dry^~^
15	Colour of lamina adaxial surface prior to drying^~^
16	Colour of lamina abaxial surface prior to drying^~^
18*	Style colour pre anthesis^~^
19*	Style colour post anthesis^~^

~=RHS colours, see Table 3. * = new characters; i.e. not previously used in studies of *Banksia* (cf. Thiele and Ladiges 1996).

Colours, however, were treated as multistate characters to maximise accuracy and repeatability, which allowed for some natural variation, thus avoiding spurious over-precision (see below). Royal Horticultural Society (RHS) colours were used to compare adaxial and abaxial leaf surfaces prior to, and after drying, as well as styles before and after anthesis. Each RHS colour was allocated a number from 1–26 (Table 3).

**Table 3.** RHS colour codes used in phenetic analysis.

Colours	RHS colours	Coded RHS colours
Green Group	135a–d	1
Green Group	137a–d	2
Yellow Green Group	146a–d	3
Yellow Green Group	147a–d	4
Greyed White Group	156a–	5
Greyed White Group	156b-d	6
Greyed White Group	157a–d	7
Greyed Green group	190a-c	8
Greyed Green Group	190d	9
Greyed Yellow Group	160a	10
Greyed Yellow Group	162a–d	11
Yellow green group	148d	12
Green White group	157b	13
Red Purple Group	59a–d	14
Red Purple Group	61a–d	15
Black Group	202a–d	16
Greyed Yellow group	160b-160d	17
Greyed Green Group	191a–d	18
Greyed Green Group	195a–d	19
Greyed Green Group	196a–d	20
Green Group	139a–d	21
Greyed Green Group	198a–d	22
Yellow Green Group	148a–d	23
Yellow Green Group	145a-d	24
Yellow Green Group	152a-d	25
Greyed Green Group	198a-d	26

All leaf measurements were taken from leaves in the middle of a branchlet, selected from the whorl of branchlets subtending a resting terminal bud or conflorescence; leaves were measured after drying. Conflorescence characters such as number of floral pairs were counted live on the plant. Infructescences were measured vertically with a steel ruler and the circumference was measured with a sewing tape measure.

Infructescences were placed on a gas burner for 1–3 min then left on brown paper for two days in a dry place. Seeds were extracted using a pair of forceps and measured under a stereomicroscope using a calibrated eyepiece graticule.

### Dataset

A dataset (Appendix 1) was maintained in Microsoft Excel and exported to PATN v. 3 for Windows (Belbin and Collins 2006). The characters were range-standardised and a distance matrix calculated using the Gower distance metric (Wills et al. 2000). Three-dimensional ordination plots were generated from the distance matrix using semi-strong hybrid multidimensional scaling (SSH MDS) with 100 random starts and 200 iterations to minimise stress. Flexible UPGMA (Beta-value = -0.1) phenograms, 3D ordination scatter plots, and correlation of characters with ordination pattern (PCC) were produced directly within PATN. The criteria for circumscribing distinct taxa were: (1) the OTUs representing the putative taxa formed discrete groups that did not overlap those of any other groups of OTUs in both cluster and ordination analysis and (2) the OTUs within these groups showed an amount of morphological heterogeneity similar to that of the other putative species in the *Banksia spinulosa* complex included in the analysis (Plunkett et al. 2009). In total 23 characters were used, 11 morphometric, five binary, seven multistate qualitatively coded morphological characters (Table 2).

## Diagnostic qualitative morphological characters

### Conflorescences

The conflorescences of all taxa in the *Banksia spinulosa* species complex consist of an elongate woody rachis that has three types of bracts. Below the base of the rachis on the short peduncle are the involucral bracts. The second type of bract is the common bract each of which subtends a flower pair on the conflorescence axis. The third type of bract, a smaller floral bract, subtends each flower in a pair (Johnson and Briggs 1975; George 1981; Thiele and Ladiges 1996). In the early stage of conflorescence development, flower pairs start to develop along the rachis basipetally. The flowers emerge from each side of the floral bracts and above and below each large common bract. Bracts and flower pairs are arranged in vertical columns on the rachis. This pattern is visually enhanced with the development of styles. The vertical striping pattern remains until the perianth and the styles have senesced or fallen from the rachis (George 1981; Thiele and Ladiges 1996; Collins et al. 2009).

### Structure of the perianth (floral pairs)

The perianth segments or tepals in *Banksia* each consist of a limb and a claw (Thiele and Ladiges 1996). In *Banksia* andmost other Proteaceaethe perianth is made up of four tepals (Wrigley and Fagg 1989; Weston 2006).

### Structure of the style

The conflorescences in the *Banksia spinulosa* complex have the appearance of being a particular colour, i.e. black, red, yellow orange, or purple. It is the styles that are most boldly coloured with red, black, green, yellow or purple pigment, not the limb and claw (George 1981; Collins et al. 2009). All styles in the *Banksia spinulosa* species complex are hooked and extend up to 3 mm past the limb and claw just prior to anthesis. The distal part of the style is modified as a pollen presenter and the stigmatic cavity is located at the apex of the style. The style is released from the limb upon anthesis (Thiele and Ladiges 1996; Weston 2006). All styles in the *Banksia spinulosa* complex have similar surfaces. Scanning electron microscopy was performed on the style surfaces and no distinguishing features were found.

### Leaf morphology

All taxa within the *Banksia spinulosa* complex have leaves that are scleromorphic in texture, discolourous, and linear in shape. The indumentum on the abaxial leaf surface is felted and the midvein is raised on the abaxial surface of all leaves in all taxa within the complex. Continuous variation was found in the colour of adaxial and abaxial leaf surfaces both within and between populations of all taxa within the *Banksia spinulosa* complex.

### Lignotubers

The term lignotuber refers to a woody swelling which may take the form of an extensive subterranean lignotuber, basal lignotuber, or an above ground lignotuber (Mibus and Sedgley 2000). The development of a lignotuber is considered to have evolved repeatedly in different lineages in response to increased fire frequency (Whelan and York 1998).

## Results and discussion

### Phenetic analysis

**Figure 1.**
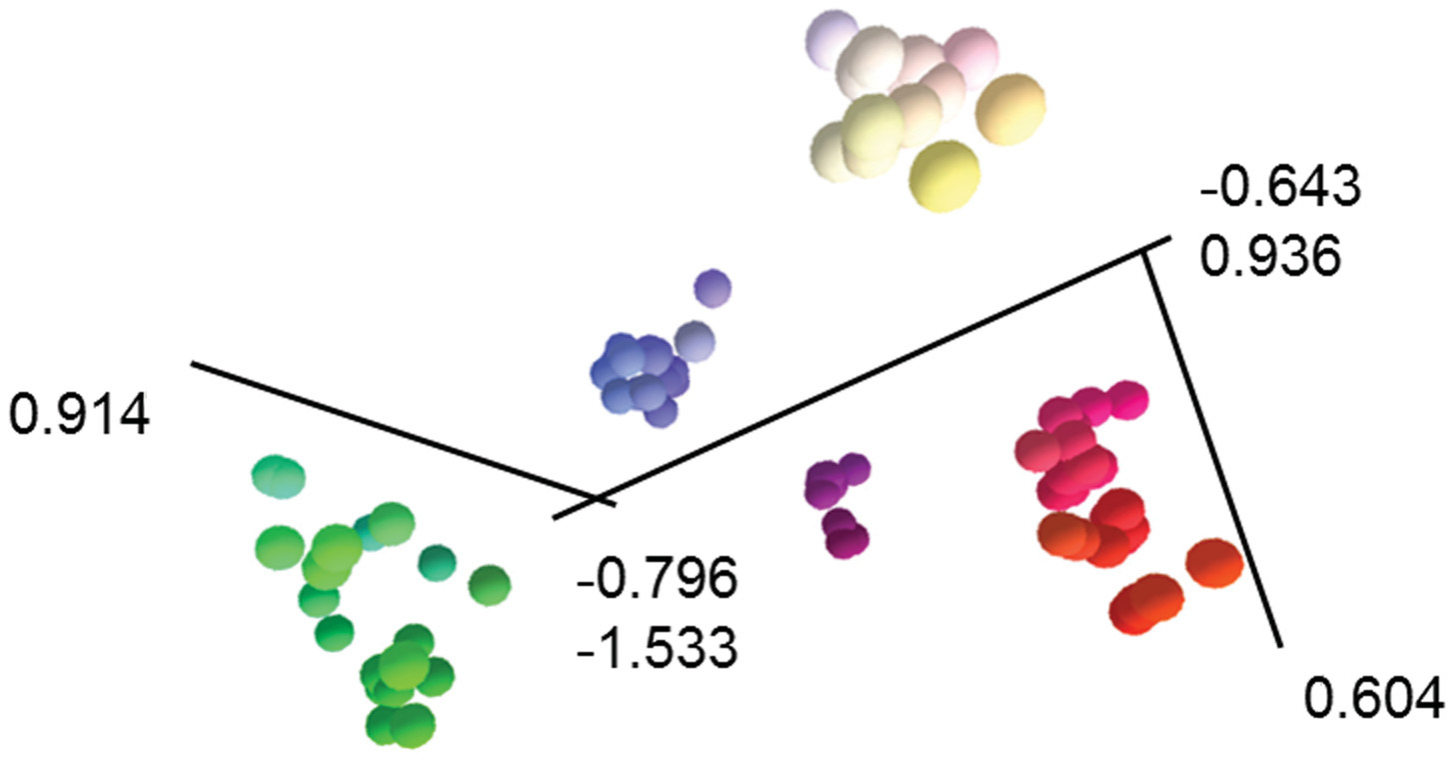
3D ordination from semi-strong multidimensional scaling of the *Banksia spinulosa* complex. From to leftto right, *Banksia spinulosa* var. *collina* sens. lat., *Banksia spinulosa* from Morisset, Bouddi and Calga**,** *Banksia spinulosa* var. *spinulosa*, *Banksia cunninghamii* subsp. *cunninghamii*, *Banksia cunninghamii* subsp. A. Ordination stress = 0.795. Size and colour of OTUs represents perspective. Ordination orientated to highlight separation of groups of OTUs. See Table 2 for characters and Appendix 1 for data.

**Figure 2.**
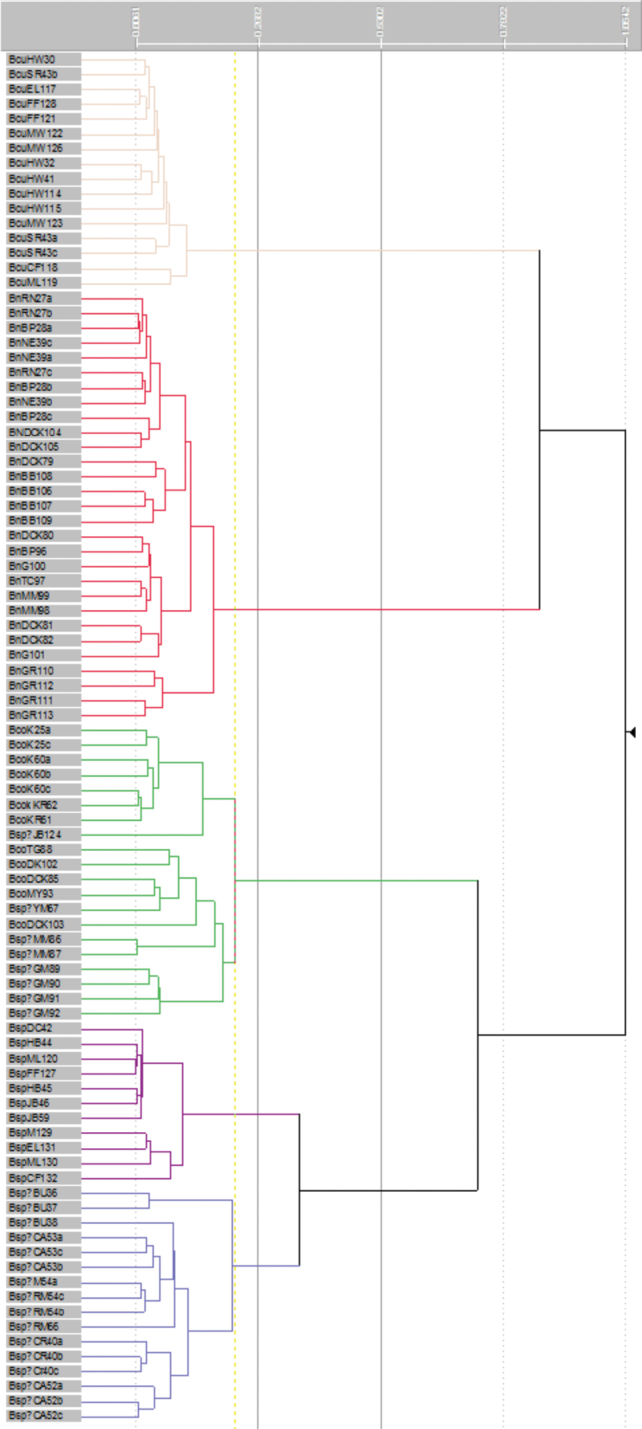
Flexible UPGMA phenogram of OTUs in the *Banksia spinulosa* complex. Major groups from top to bottom: *Banksia cunninghamii* subsp. *cunninghamii*, *Banksia cunninghamii* subsp. A, B. *spinulosa* var. *collina* sens. lat., *Banksia spinulosa* var. *spinulosa*, *Banksia spinulosa* from Morisset, Bouddi and Calga. See Table 2 for characters and Appendix 1 for data.

Ordination (Figure 1) and clustering (Figure 2) of the data matrix found five distinct groups of OTUs in the *Banksia spinulosa* complex: corresponding to *a priori* names *Banksia spinulosa* var. *collina* *sens. lat*., *Banksia spinulosa* var. *collina* **×** *Banksia spinulosa* var. *spinulosa* from near the New South Wales locations of Morisset, Bouddi and Calga, *Banksia spinulosa* var.* spinulosa*, *Banksia cunninghamii* subsp. *cunninghamii, B. cunninghamii* subsp. A. The phenogram displays the same five groups of OTUs (Figure 2). Even when we reran the analyses excluding all the binary characters (Characters 11, 20–23; ordination and phenogram not presented), the same five groups of OTUs were obtained, which, along with the very low stress value (Figure 1) indicate that the results are robust. Twelve of the 23 characters, including quantitative, binary and multistate characters had correlated more than 70% with the ordination (Table 4) indicating sound choice of characters, a broad base of evidence for the patterns obtained, and confidence in the results obtained.

**Table 4.** Principal component correlation (PCC) attributes and ordination vectors for ordination of the *Banksia spinulosa* complex. See Table 2 for Character numbers.

**Character**	**X**	**Y**	**Z**	**Correlation (r2)**
21	-0.115	0.983	0.146	0.978
20	0.35	0.67	0.655	0.953
14	-0.254	0.695	0.672	0.938
23	-0.604	0.473	-0.641	0.938
18	-0.464	0.482	-0.743	0.929
13	0.872	0.195	-0.45	0.917
22	-0.787	-0.327	0.523	0.893
11	-0.534	-0.77	-0.35	0.847
8	0.767	0.269	-0.583	0.84
9	-0.678	0.6	-0.425	0.826
10	0.146	0.72	0.679	0.766
19	-0.104	0.415	-0.904	0.732
3	-0.477	0.868	0.141	0.581
6	0.348	0.868	-0.355	0.537
2	-0.339	0.662	-0.669	0.454
1	-0.47	0.702	-0.535	0.413
16	0.174	-0.25	-0.952	0.391
15	0.45	0.036	-0.892	0.372
12	-0.326	-0.601	0.729	0.308
7	0.433	0.858	-0.277	0.279
5	0.005	0.635	-0.772	0.193
4	0.714	0.694	0.092	0.146
17	-0.614	0.567	-0.549	0.123

The cluster of OTUs of *Banksia spinulosa* from Morisset, Bouddi and Calga (Table 1) is characterised by red styles, at Morisset and Bouddi and black styles at Calga, multi-stemmed habit and occurs between the Hawkesbury River and Hunter Valley. Herbarium specimens from these locations have been determined by A.S. George and other botanists as “*Banksia spinulosa* var. *collina* **×** *Banksia spinulosa* var. *spinulosa*”. George (1981) considered this group of OTUs to be an intergrade between *Banksia spinulosa* var. *spinulosa* and *Banksia spinulosa* var.* collina*. These populations do not fall in a position intermediate between *Banksia spinulosa* var. *collina* and *Banksia spinulosa* var. *spinulosa* in the ordination diagram, nor do they segregate into three clusters representing parental species and hybrids. There is thus no clear phenetic evidence of either an intergrade or a mixture of hybrids and parental species between the Hawkesbury River and Hunter Valley. The taxonomic status of these populations and their relationships to others remains unclear. This cluster of OTUs could represent a distinct species, but we will investigate this question and the broader relationship between *Banksia spinulosa* var. *collina* and *Banksia spinulosa* var. *spinulosa* further before making any formal taxonomic changes to these taxa.

Slight outliers in the *Banksia spinulosa* var. *collina* cluster represent some discontinuous morphological variation, which we also plan to investigate.

### Taxonomic inference

Given the consistent clear cut groups in the ordination and cluster analysis across a broad geographic and morphological range of OTUs (Table 1), we propose the following taxonomic arrangement, which we use hereafter in this paper: recognising *Banksia cunninghamii* subsp. *cunninghamii* as *Banksia cunninghamii* *sensu stricto*; recognising *Banksia spinulosa* var. *collina* as *Banksia collina* *sensu lato*; recognising *Banksia spinulosa* var. *spinulosa* as *Banksia spinulosa* *sensu stricto*; formalising *Banksia cunninghamii* subsp. A at species rank under the name *Banksia neoanglica* . Although the OTUs of *Banksia spinulosa* from the Morisset and Bouddi populations could be considered to constitute a distinct species on the evidence we present here, we refrain from recognising these populations as a distinct taxon until we have more thoroughly tested the hypothesis that they are part of an extensive hybrid swarm and searched for any additional populations that might provide evidence for integradation between *Banksia collina* and *Banksia spinulosa*.

## Morphological analysis

### Growth forms within the *Banksia spinulosa* species complex

*Banksia cunninghamii* *sensu stricto* is a single-stemmed tree to 7 m tall, and is non-lignotuberous. *Banksia spinulosa* *sensu stricto* forms a multi-stemmed, rounded shrub to 3 m high. The lignotuber is subterranean. *Banksia collina* *sensu lato* is a multi-stemmed upright shrub to 3 m tall, with a subterranean lignotuber (Harden 2002; George 1981).

*Banksia neoanglica* has a variety of growth forms ranging from small rounded multi-stemmed shrubs to single-stemmed trees. The growth forms of *Banksia neoanglica* appear to be related to the degree of exposure of plants to fire. At sites where there have been no fires for more than 15 years, such as at Binna Burra, Lamington National Park, Queensland and some parts of Gibraltar Range National Park, New South Wales (Pers. Comm. Justin Kreis Ranger Glen Innes National Park), *Banksia neoanglica* is a single-stemmed tree and exhibits all the traits of an obligate seeder such as a greater infructescence load and spontaneous opening of the follicles. In the tree form, *Banksia neoanglica* has a slight swelling at the base of the trunk just below the soil or there are epicormic buds which often develop into branches, well above ground level, similar to those of some eucalypts (Burrows 2008). The multi-stemmed form has a substantial subterranean lignotuber and requires fire to open follicles and has a greatly reduced infructescence load.

### Individual adult morphological features

**Styles:** The structure of the conflorescence, including perianth and styles is similar for all taxa in the *Banksia spinulosa* complex. Size, shape and colour of the individual parts of the conflorescence, however, differ considerably across the species. Style colour in the *Banksia spinulosa* complex varies depending on the proportions of chlorophyll (green), carotenoid (yellow to orange), anthoxanthin (yellow) and anthocyanin (red to purple) pigments that develop in them (Grotewold 2006). The style colour in *Banksia neoanglica*, *Banksia spinulosa* *sensu stricto* and *Banksia cunninghamii* *sensu stricto* usually grades from red to maroon to purple during conflorescence development, then the style becomes discolorous at anthesis, with the apex becoming dark purple to black. This is a consistent character within and between populations of three species in the *Banksia spinulosa* complex. The exception is *Banksia collina* sensu lato which has concolourous green styles both before and after anthesis. We found no black-styled *Banksia collina* *sensu lato* within the geographical range of this project.

The style apex *in B. cunninghamii sensu stricto* seems to have substantially more anthocyanin pigment than either *Banksia spinulosa* *sensu stricto* or *Banksia neoanglica*. In *Banksia cunninghamii* *sensu stricto* we observed that the style length is usually longer than either *Banksia spinulosa* *sensu stricto* or *Banksia neoanglica* and is a similar length to *Banksia collina* *sensu lato*. The black pigmentation of the styles of *Banksia cunninghamii* *sensu stricto* starts to develop one third of the way along the style above the ovary. In *Banksia spinulosa* *sensu stricto* and *Banksia neoanglica* the dark pigmentation in the style develops one half to two thirds of its length above the ovary. In all populations in the *Banksia spinulosa* complex with the exception of *Banksia collina* *sensu lato* we observed what appeared to be yellow-styled conflorescences. Upon closer inspection they are green styled and appear to have less chlorophyll in both the styles and leaves than is found in *Banksia collina* *sensu lato* which is also green-styled. Green styled variants are found in less than 2% of any one population except in *Banksia collina*. Polymorphism is a common trait in Proteaceae where, for example, 40% of all species of *Protea* exhibit variation in the bract, style and perianth colour (Carlson et al. 2010). It is often unclear whether these variants are transient mutant individuals or this feature is a persistent polymorphism (Carlson et al. 2010). In the case of the *Banksia spinulosa* complex, however, the variants comprise less than 2% of a population and were not found in every population; therefore it is unlikely to be persistent polymorphism.

**Perianth colour:** The colours of the perianth in the *Banksia spinulosa* complex vary according to their developmental stage and their exposure to sunlight. The perianth colours can vary within and between populations in all four of the species in the *Banksia spinulosa* complex. The factor that seems to have the most influence on the perianth colour in the early stages of development is exposure to sun, often mediated by the position of an conflorescence on the outside or inside branches of the plant or by shading from other plants. In *Banksia spinulosa* *sensu stricto*, *Banksia collina* and *Banksia neoanglica*, the conflorescences that are exposed to full sun tend to have orange or yellow perianths. Those that are exposed to a limited amount of sun tend to be green. The perianth colour of *Banksia cunninghamii* *sensu stricto* is diagnostic for the species. At maturity the perianth always has a distinct pink hue and this colouring continues through to anthesis. The pink hue does not vary between and within populations of *Banksia cunninghamii* *sensu stricto*, nor does exposure to full sun orfull shade effect the colour of the perianth at maturity.

**Figure 3.**
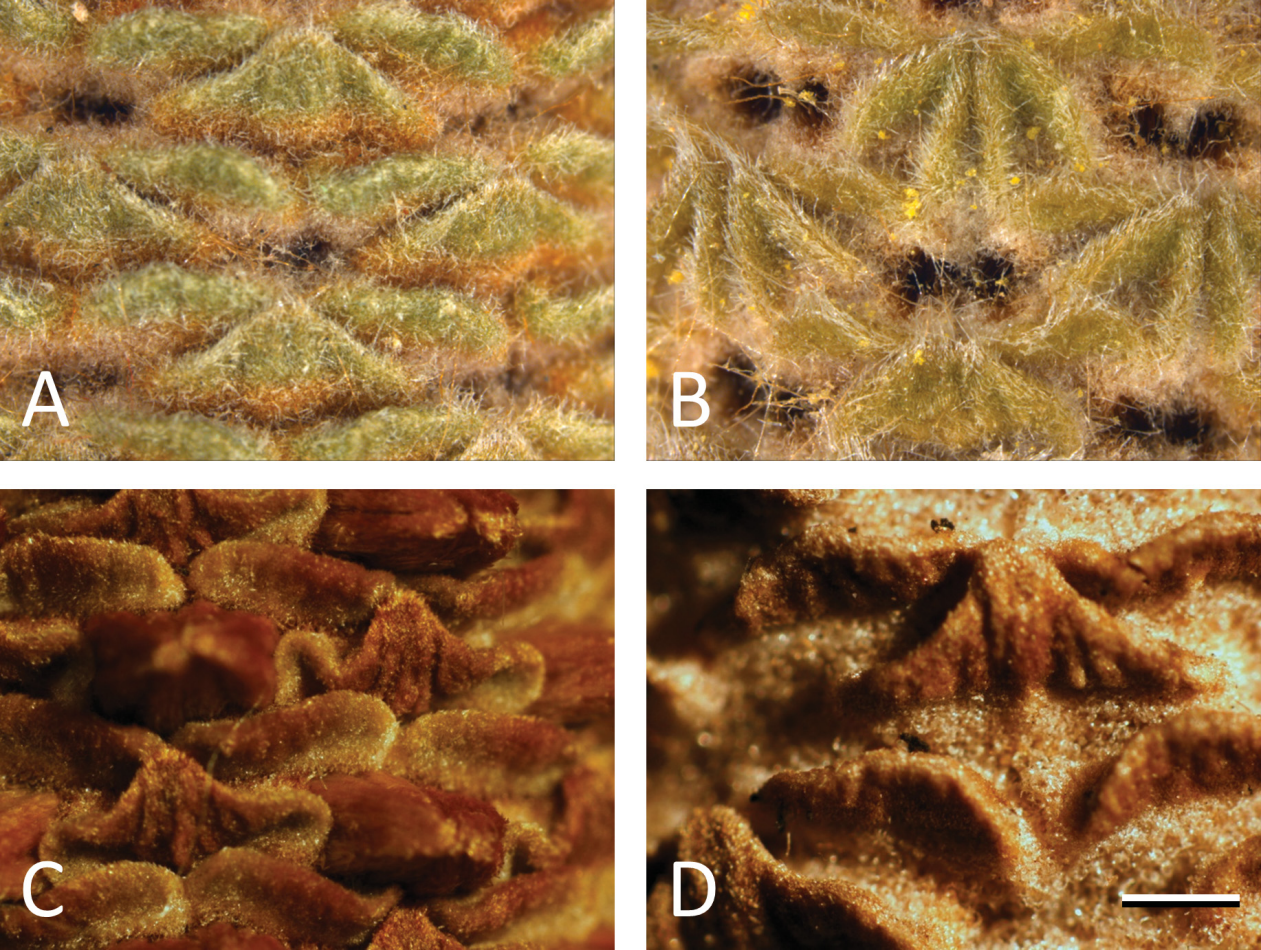
Common bractson young conflorescences in the *Banksia spinulosa* species complex: **A** *Banksia neoanglica* (*BanksiaL. Stimpson 98*) **B** *Banksia cunninghamii* *sensu stricto* (*M.L. Stimpson 122*) **C** *Banksia collina* *sensu lato* (*M.L. Stimpson 25A*) **D** *Banksia spinulosa* *sensu stricto*. (*M.L. Stimpson 120*). Scale bar = 1 mm.

**Common bracts:** Common bracts have been mentioned in previous studies (Johnson and Briggs 1975; Thiele and Ladiges 1996; George 1981) but the bract surfaces had not been mentioned before this study or used to draw taxonomic conclusions. Close examination, especially at early stages of development, of the abaxial surface of the common (or flower pair) bracts found them to have differences in shape, texture, colour, and surface (Figure 3A–D) which covary in line with the entities recognised here (Figures 1–2) within the *Banksia spinulosa* complex. We will characterise these differences for use in future expanded phenetic analysis and description of taxa in *Banksia*. Floral bracts were not examined in detail in this study.

**Figure 4.**
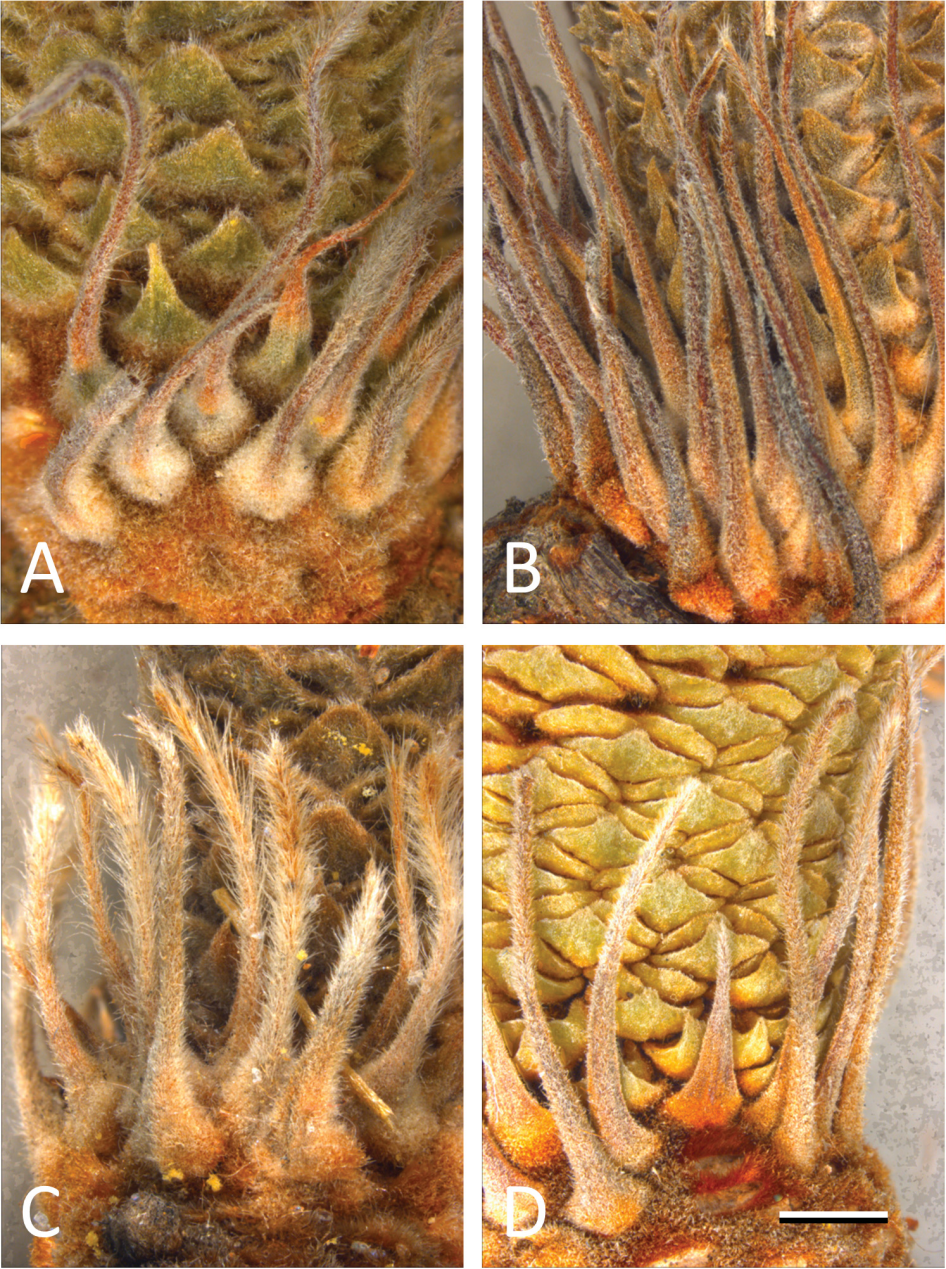
Involucral bracts on young conflorescences in the *Banksia spinulosa* complex. **A** *Banksia cunninghamii* *sensu stricto* (*M.L. Stimpson 122*) **B** *Banksia spinulosa* *sensu stricto* (*M.L. Stimpson 125*) **C** *Banksia neoanglica* (*M.L. Stimpson 81*) **D** *Banksia collina* *sensu lat*o (*M.L. Stimpson 25A*). Scale bar = 2.5 mm.

**Involucral bracts:** Involucral bracts appear to be taxonomically informative at the species level in the study group. The involucral bracts of *Banksia cunninghamii* *sensu stricto* are caudate with an abaxial ‘spine’ (Figure 4A). The involucral bracts in *Banksia spinulosa* *sensu stricto* (Figure 4B) are longer and more scleromorphic, with little or no hair and no external spine. In *Banksia neoanglica* (Figure 4C) these bracts are more hirsute without an external spine and in *Banksia collina* *sensu lato* (Figure 4D) the involucral bracts are shorter, have no external spine and limited hair. There are differences in the distal and proximal portions of the involucral bracts (Figure 4A–D) in each species that warrant further examination.

## Taxonomic conclusions

The diversity of species concepts in the biological literature is an asset, not a liability when considering the *Banksia spinulosa* complex and are an integral part of biological theory. We have taken into account the co-varying morphological discontinuities, the phenetic species concept, geographical and ecological isolation and the biological species concept of reproductive isolation. The use of differing concepts has been useful in suggesting multiple lines of evidence for testing taxonomic boundaries in the *Banksia spinulosa* complex (cf. de Queiroz 2007). Clear taxonomic groups were obtained based on the results of the morphometric analyses and corroborated by new characters (cf. Thiele and Ladiges 1996) such as the abaxial surface of the common bract (Figure 3), the number of floral pairs around the circumference of the conflorescence and obvious differences in the involucral bracts (Figure 4A–D). Additionally, given the ecological isolation, reproductive isolation and morphometric differentiation of at least three of the taxa in the *Banksia spinulosa* complex, there is a compelling case to recognise *Banksia spinulosa* *sensu stricto*, *Banksia cunninghamii* *sensu stricto* and *Banksia neoanglica* as separate species (Table 5). *Banksia collina* *sensu lato* is considered heterogeneous and in need of further study, but is not readily confused with *Banksia neoanglica*. Similarly *Banksia spinulosa* from the Morisset, Bouddi and Calga requires further study but is distinct from *Banksia neoanglica*.

**Table 5.** Comparison of some attributesof *Banksia neoanglica*, *Banksia spinulosa* *sensu stricto* and *Banksia cunninghamii*.

**Character**	***Banksia neoanglica***	***Banksia spinulosa***	***Banksia cunninghamii***
Lignotuber	present	present	absent
Leaf length	43–75 mm	50–72 mm	53–88 mm
Leaf width	3–4.5 mm	1.5–2.5 mm	3–4.5 mm
Leaf margins	not recurved	tightly recurved	not recurved
Length of inflorescence	84–119 mm	96–144 mm	99–152 mm
Common bract keels	single thickened keel	single keel apex	two thin keels
Common bract apex	apex rounded	apiculate	apex rounded
Number of floral pairs	12–14(–16) pairs	13–16 pairs	12–14 pairs
Perianth colour	orange, or yellow	orange or yellow	pink
Style colour prior to anthesis	red/maroon	red/maroon	red/maroon
Style colour after anthesis	purple/black	purple/black	purple/black
Circumference of infructescence	141–160 mm	153–159 mm	113–125 mm
Length of infructescence	85–120 mm	96–144 mm	113–140 mm

The geographic distribution of *Banksia neoanglica* falls within the biogeographic region known as the “Macpherson–Macleay Overlap” of Burbidge (1960), which is a biogeographically distinctive and rich area (Crisp et al. 1999) with many species of plants and invertebrates endemic to the area.

*Banksia cunninghamii* *sensu stricto* and *Banksia neoanglica* have often been misidentified because *Banksia cunninghamii* *sensu stricto*, on occasions, has a brown indumentum; *Banksia neoanglica* sometimesalso exhibitsbrowning on the abaxial leaf surface. This character has been used in the past as an aid to distinguishing *Banksia cunninghamii* *sensu stricto* and the two other ‘varieties’ recognised at that time (George 1981; Harden 2002). Indeed, this attribute occurs in both *Banksia neoanglica* and *Banksia cunninghamii* *sensu stricto*. Drying of the specimens in both of these species can cause browning on the abaxial leaf surface. The browning of the abaxial leaf surface should not be used as taxonomic marker or an identification tool.

### Future directions

Disjunct populations in central and northern Queensland currently assigned to *Banksia spinulosa* var. *spinulosa* warrant inclusion in a more broadly framed analysis, as do the northern and southern populations of *Banksia collina* *sensu lato* and Victorian populations of *Banksia cunninghamii* *sensu stricto*. There are also other populations of *Banksia* that clearly belong with the *Banksia spinulosa* group but are as yet unstudied. Further work is needed to enable suitable placement of these populations. Analysis using molecular data, together with expanded use of the novel characters presented here, would likely resolve these long-outstanding taxonomic issues.

## Taxonomic treatment

### Banksia neoanglica

(A.S.George) Stimpson & J.J.Bruhl stat. nov.

http://species-id.net/wiki/Banksia_neoanglica

Banksia spinulosa Sm. var. *neoanglica* A.S.George, *Nuytsia* 6: 315 (1988).

#### Type.

**Figure 5.**
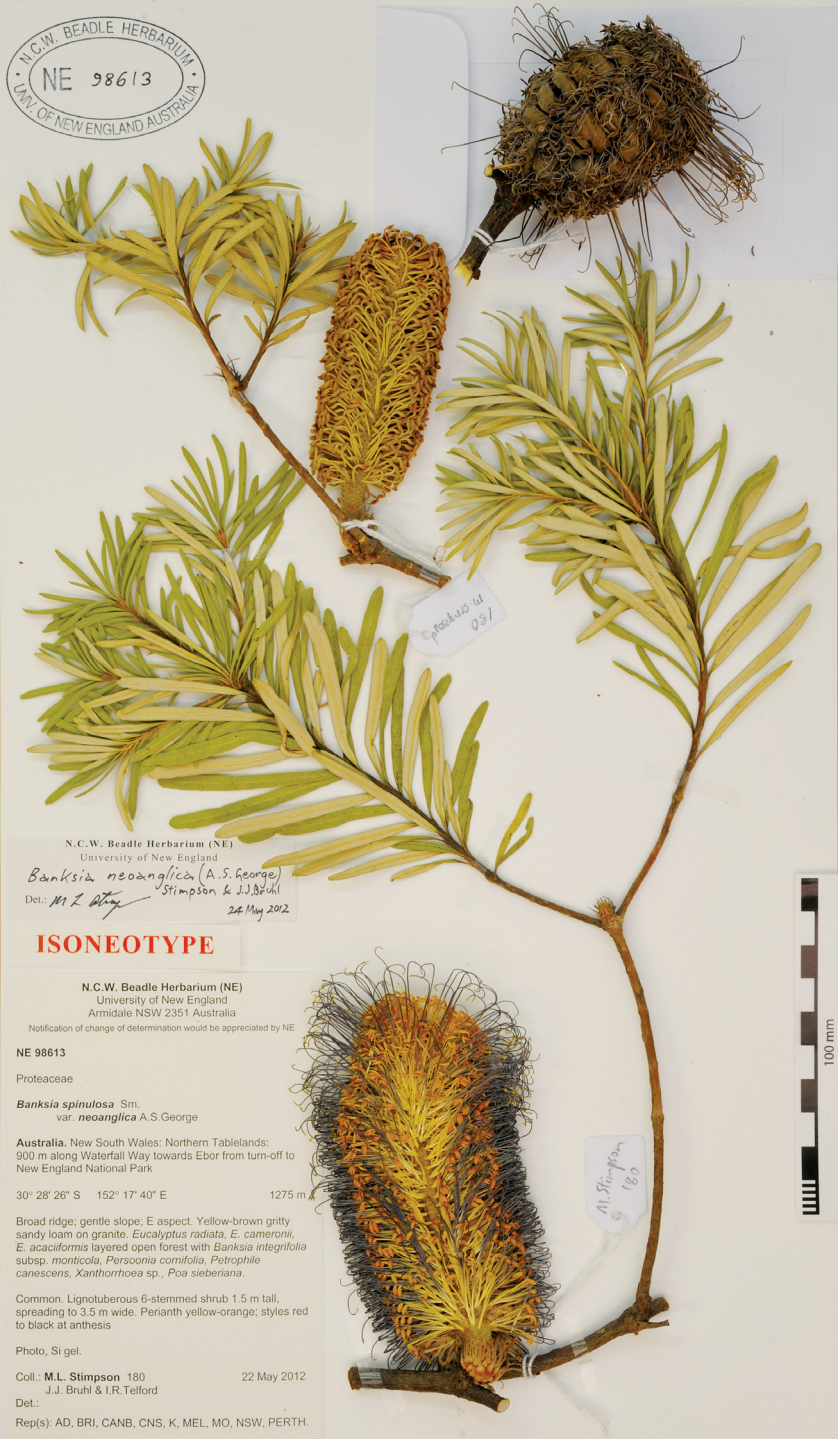
Photograph of the neotype of *Banksia spinulosa* var. *neoanglica* A.S.George (*M.L. Stimpson 180, J.J. Bruhl & I.R. Telford*, NE 98613).

AUSTRALIA: New South Wales: Northern Tablelands, 900 m along Waterfall Way towards Ebor from turn-off to New England National Park, 22 May 2011, *M.L. Stimpson 180, J.J. Bruhl & I.R. Telford*; neotype: NSW; isoneotype: AD, BRI, CANB, CNS, K, MEL, NE, MO, PERTH. Figure 5.

*Banksia spinulosa* Sm. var. *cunninghamii* (Sieber ex Rchb.) A.S.George, *Nuytsia* 3: 396 (1981) *pro parte*, excluding type.

*Banksia cunninghamii* Sieber ex Rchb. subsp. A: G.J. Harden in G.J. Harden (ed.), *Flora of New South Wales* 1: 71 (1991); G.J Harden, D.W. Harden & D.C. Godden (2000) *Proteaceae of New South Wales*: 170 (2000); G.J. Harden in G.J. Harden (ed.), *Flora of New South Wales* 2, edn 2: 86 (2002).

The protologue of *Banksia spinulosa* var. *neoanglica* quotes the type:

“1 km N of turnoff to New England National Park, Ebor–Armidale road, N.S.W., 6 April 1986, *S.C. Clemesha*; holo: NSW; iso: CANB, BRI, MEL, PERTH”.

No specimens so labelled have been located in NSW, BRI, CANB or MEL herbaria after repeated searches. Alex George (pers. comm. 2010–2011) could find no specimens in PERTH and he believes it likely that specimens were never distributed. Accordingly, we have nominated a neotype, collected from the same population as the type.

#### Description.

**Figure 6.**
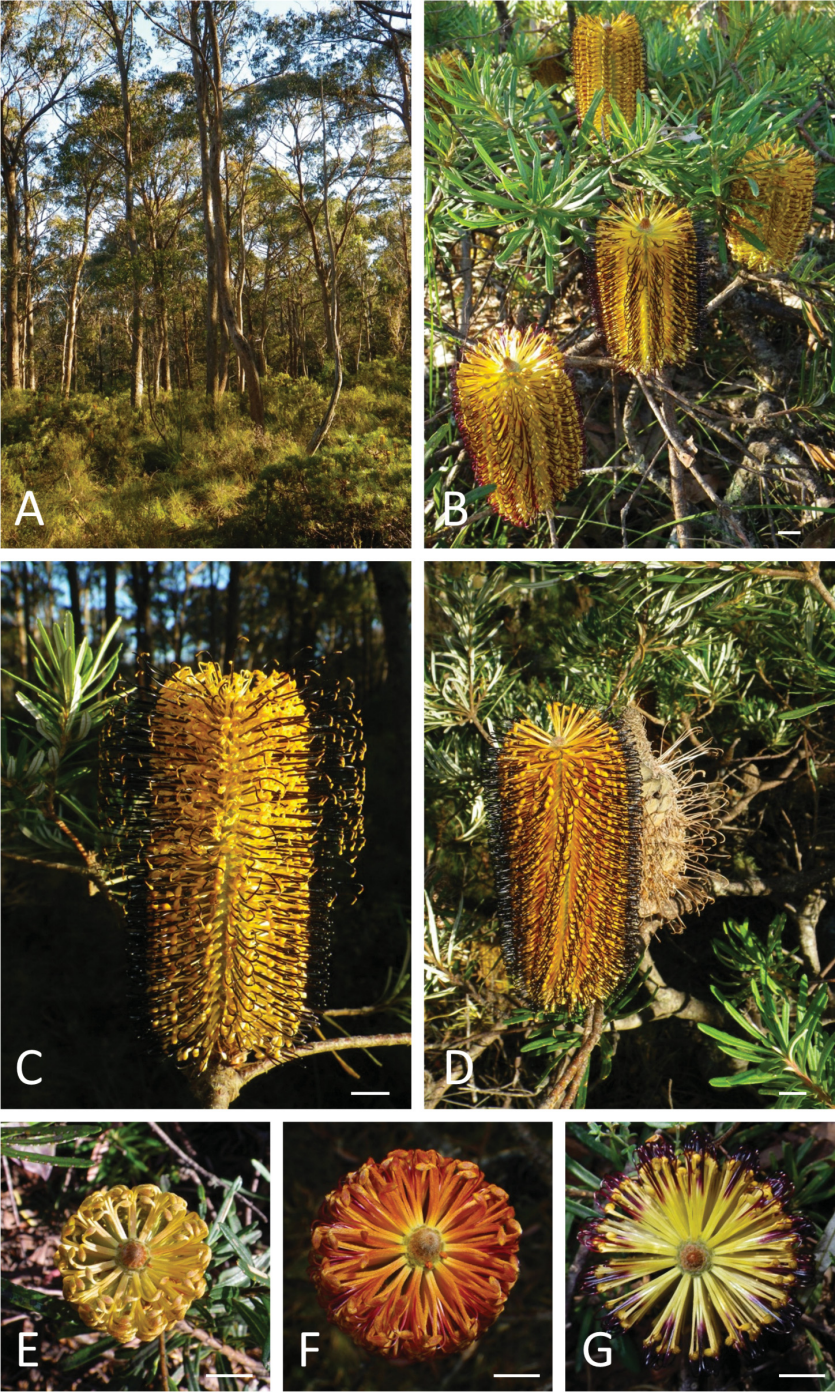
*Banksia neoanglica* at neotype locality. **A** Habitat **B** Conflorescences on shrub **C** Conflorescence from the neotype collection(*M.L. Stimpson 180, J.J. Bruhl & I.R. Telford*) showing basipetal development; upper flowers with pollen on pollen presentors **D** Conflorescence and infructescence with black styles at preanthesis. **E–G** Apex of conflorescences at successive stages of development exhibiting variation in perianth and style colour. Scale bars = 1 cm.

Shrubs with 2–8(–10) stems to 2.5 m from a lignotuber or trees to 7 m tall. Juvenile leaves: petiole 2–3.8 mm long; lamina narrowly obovate, 30–66 mm long, 5–11 mm wide, strongly dentate along full leaf margin, apex bidentate. Adult leaves: petiole 1.8–3.5 mm long; lamina linear, 43–75 mm long, 3–4.5 mm wide, occasionally toothed towards the usually unidentate, occasionally bidentate apex; adaxial surface glabrous, with colour after drying RHS greyed green group 195a-d; abaxial surface felted, colour after drying RHS greyed white group 156a–d. Involucral bracts subulate, thickened at base, 3–15mm long, grey-brown pubescent. Conflorescence 84–119 mm long, 70–85 mm diameter at anthesis; floral pairs 12–14(–16) around the circumference of the conflorescence axis. Common bract with a single thickened keel on the abaxial surface that extends from the apex of the bract down to the visible part of the base of the bract, distal margins slightly concave, apex rounded, indumentum villous, lower third of bract uniformly brown and upper two thirds uniformly green (Fig. 3A). Perianth 18–23 mm long, pubescent, yellow–orange at maturity but may be green, orange or yellow during developmental stages; limb c. 3.5 mm long; anthers c. 1 mm long. Style 25–38 mm long, apically hooked, colour grading from red to maroon to black just prior to anthesis. Infructescence 85–120 mm long, 35–45mm diam. Seed 15–19 mm long, including wing. Figure 6.

#### Distribution.

**Figure 7.**
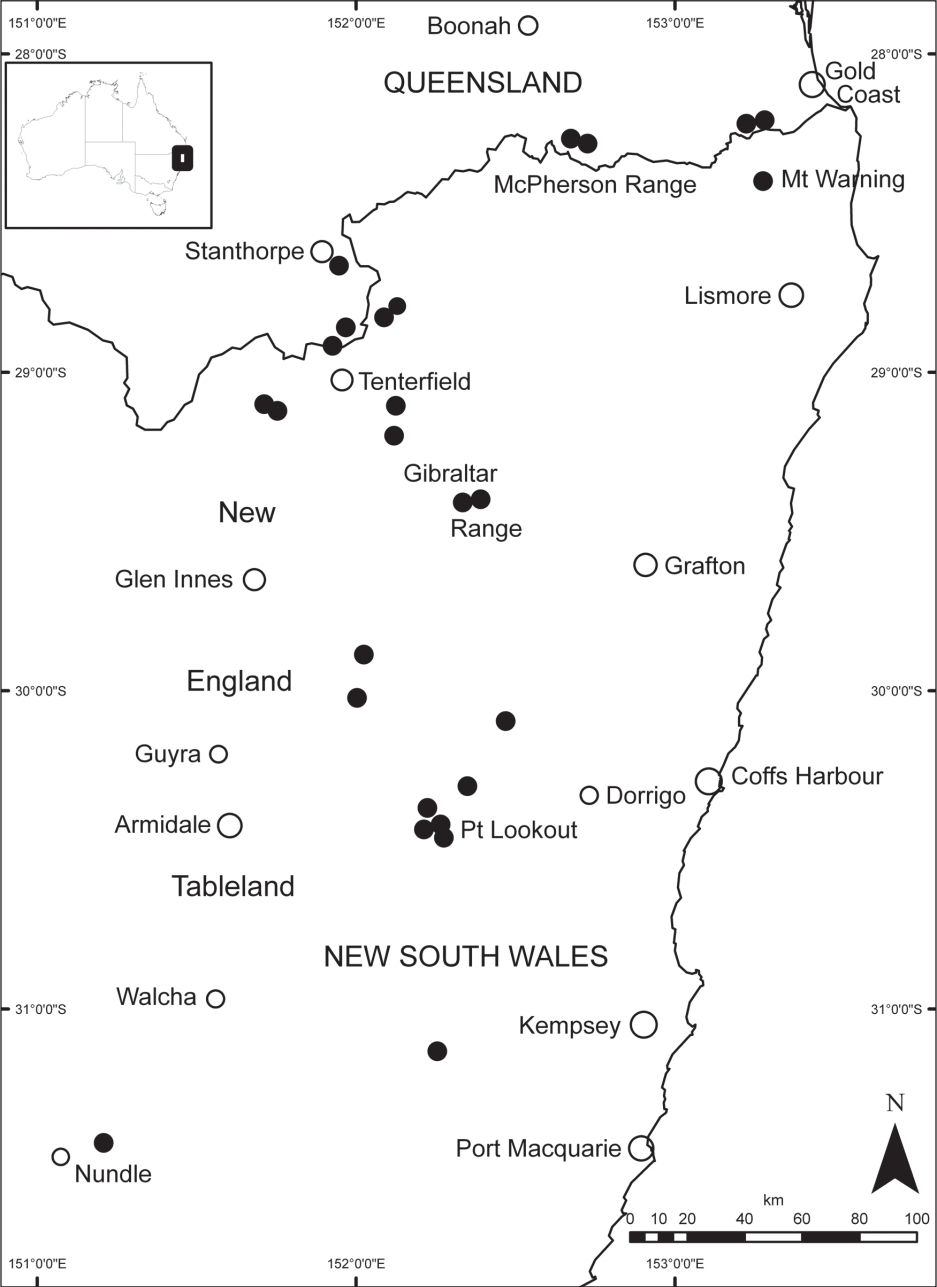
Distribution of *Banksia neoanglica*(solid black circles). Towns and cities indicated by open circles.

*Banksia neoanglica* occurs on the McPherson Range, just north of the Queensland–New South Wales border, Mt Warning and the eastern edge of the New England Tableland southwards to near Hanging Rock, New South Wales. Figure 7.

#### Ecology.

Grows in sandy soil on granite and acid volcanics, rarely on basalt, in *Eucalyptus* open forest (Figure 6), woodland and heath at altitudes of 850–1480 m. The species is sympatric with *Banksia integrifolia* subsp. *monticola* throughout its range, with *Banksia marginata* *sensu lato* on the Gibraltar Range and with *Banksia conferta* in the Daves Creek area.

The growth forms that *Banksia neoanglica* assume appear to be dependent upon the exposure to fire (Whelan and York 1998). In areas where there have been no fires for more than 15 years, such as Lamington National Park, Queensland, and some parts of Gibraltar Range, New South Wales (pers. comm. Justin Kreis 25 May 2010), a single-stemmed habit is found. Here, the lignotuber is present as a stem thickening just above or just below the soil surface, and branchlets may sprout from epicormic buds up to 30 cm above the ground. This single-stemmed form of *Banksia neoanglica* behaves like an obligate seeder with a heavy infructescence load and follicles open spontaneously without fire. More commonly the plants are multi-stemmed, with up to 2–8(–10) stems from a subterranean lignotuber carry a much lower infructescence load, usually 1–3(–5) infructesences per plant. Fire is required to open the follicles**.**

#### Conservation status.

The species is widespread, often locally common, and is not considered at risk. It is conserved in several reserves: Lamington, Springbrook and Girraween National Parks in Queensland, and Boonoo Boonoo, Gibraltar Range and New England National Parks and Torrington State Conservation Area in New South Wales.

#### Selected specimens examined.

AUSTRALIA. Queensland: Moreton District: McPherson Range, Lamington National Park, Daves Creek track, *M.L. Stimpson 79* (BRI, NE, NSW); Darling Downs District: Girraween National Park, track to Mt Norman, 21 Jan. 2009, *I.R. Telford 13278 & J.J. Bruhl* (NE). New South Wales: North Coast: Mount Warning, 3 Oct. 1939, *F.A. Rodway s.n*. (NSW); Northern Tablelands: 19 km E of Deepwater on Miles Shaw Rd, Butterleaf State Forest, *J.T. Hunter 3750 & P.J. Clarke* (NE); ); 0. 4 km N of Torrington, 19 Nov 1972, *J.B. Williams s.n*. (NE); Pheasant Mountain, 32 km NE of Guyra, 24 Apr. 1972, *H.J. Wissmann s.n*. (NE); Mount Chaelundi, E side just below crest, *J.T. Hunter 157 & V.H. Hunter* (NE); New England National Park, Banksia Point, *M.L. Stimpson 28* (BRI, NE, NSW); NE of Bakers Downfall Hill, Nundle State Forest, *J.R. Hosking 1877* (CANB, MEL, NE, NSW).

#### Phenology.

Resting buds start to expand in late January and conflorescences are fully developed by late March with flowering continuing until early July. These times are dependent on climatic conditions.

#### Breeding system.

Extensive experiments conducted between May 1986 and July 1987 found that the New England population of *Banksia neoanglica* studiedwas autogamous (Vaughton 1988).

## Supplementary Material

XML Treatment for Banksia neoanglica

## Appendix 1

Dataset of *Banksia spinulosa* complex used for phenetic analysis. See Table 1 for OTU codes and Table 2 for character list. (doi: 10.3897/phytokeys.14.3415.app) File format: MS Word (DOC).

**Explanation note:** The dataset (organised in MS Excel) presented here as a MS Word document, includes 23 characters and 92 individuals (operational taxonomic units = OTUs).
